# The Effects of *Andrographis paniculata* (Burm.F.) Wall. Ex Nees and Andrographolide on Neuroinflammation in the Treatment of Neurodegenerative Diseases

**DOI:** 10.3390/nu15153428

**Published:** 2023-08-02

**Authors:** Francesca Bosco, Stefano Ruga, Rita Citraro, Antonio Leo, Lorenza Guarnieri, Jessica Maiuolo, Francesca Oppedisano, Roberta Macrì, Federica Scarano, Saverio Nucera, Irene Bava, Ernesto Palma, Carolina Muscoli, Juan Hancke, Giovambattista De Sarro, Vincenzo Mollace

**Affiliations:** 1 Department of Health Sciences, Institute of Research for Food, Safety, and Health (IRC-FSH), University Magna Graecia of Catanzaro, 88100 Catanzaro, Italy; rugast1@gmail.com (S.R.); foppedisano@unicz.it (F.O.); robertamacri85@gmail.com (R.M.); federicascar87@gmail.com (F.S.); saverio.nucera@hotmail.it (S.N.); irenebava@libero.it (I.B.); palma@unicz.it (E.P.); muscoli@unicz.it (C.M.); mollace@libero.it (V.M.); 2 Section of Pharmacology, Science of Health Department, School of Medicine, University Magna Graecia of Catanzaro, 88100 Catanzaro, Italy; citraro@unicz.it (R.C.); aleo@unicz.it (A.L.); desarro@unicz.it (G.D.S.); 3 Research Center FAS@UMG, Science of Health Department, University Magna Graecia of Catanzaro, 88100 Catanzaro, Italy; 4 Laboratory of Pharmaceutical Biology, IRC-FSH Center, Department of Health Sciences, School of Pharmacy and Nutraceutical, Faculty of Pharmacy, University Magna Graecia of Catanzaro, 88100 Catanzaro, Italy; maiuolo@unicz.it; 5 HP Ingredients, Bradenton, FL 34205, USA; juan@hpingredients.com

**Keywords:** *Andrographis paniculata*, andrographolide, neurodegenerative diseases, Alzheimer’s, Parkinson’s, brain ischemia, neuroinflammation

## Abstract

Neurodegenerative diseases (NDs) affect millions of people worldwide, and to date, Alzheimer’s and Parkinson’s diseases are the most common NDs. Of the many risk factors for neurodegeneration, the aging process has the most significant impact, to the extent that it is tempting to consider neurodegenerative disease as a manifestation of accelerated aging. However, genetic and environmental factors determine the course of neurodegenerative disease progression. It has been proposed that environmental stimuli influence neuroplasticity. Some clinical studies have shown that healthy lifestyles and the administration of nutraceuticals containing bioactive molecules possessing antioxidant and anti-inflammatory properties have a preventive impact or mitigate symptoms in previously diagnosed patients. Despite ongoing research efforts, the therapies currently used for the treatment of NDs provide only marginal therapeutic benefits; therefore, the focus is now directly on the search for natural products that could be valuable tools in combating these diseases, including the natural compound *Andrographis paniculata* (Ap) and its main constituent, andrographolide (Andro). Preclinical studies have shown that the aqueous extract of Ap can modulate neuroinflammatory and neurodegenerative responses, reducing inflammatory markers and oxidative stress in various NDs. Therefore, in this review, we will focus on the molecular mechanisms by which Ap and Andro can modulate the processes of neurodegeneration and neuroinflammation, which are significant causes of neuronal death and cognitive decline.

## 1. Introduction

Neurodegenerative diseases (ND) exhibit specific epidemiological and symptomatologic characteristics and are diagnosed using separate laboratory and neuroimaging tests; the neuropathological mechanisms and treatments are also different. However, all neurodegenerative diseases can cause morbidity and reduced cognitive capacity in elderly individuals worldwide [1,2,3,4]. Alzheimer’s disease (AD) accounts for nearly 80% of all dementia cases, and [5,6,7,8,9,10] it is estimated that by 2050, the number of sufferers will reach 106.8 million, of which 16.51 million will be Europeans [11]. The incidence rate increases with age [6,12], and the annual risk reaches 6% in individuals over 85.

After AD, idiopathic Parkinson’s disease (PD) is the second most common neurodegenerative disease, with an incidence rate of 11–19/100.000 individuals per year [13] in Europe, and an average age of onset of 60 years [14,15]. In addition, the etiology of the disease in most patients is unknown. However, some genes implicated in the pathogenesis of familial forms have been identified, but this only accounts for 5–15% of cases. Pathologically, PD is characterized by the loss of dopaminergic neurons in the pars compacta of the substantia nigra and the accumulation of misfolded α-synuclein, which is found in intra-cytoplasmic inclusions called Lewy bodies. Dementia with Lewy bodies (DLB) and Parkinson’s disease exhibit many overlapping features, including progressive cognitive impairment, behavioral disturbances, and dyskinesia [16,17]. These two diseases differ, however, in the timing of the onset of motor symptoms compared to cognitive symptoms. In Parkinson’s, motor syndrome precedes the cognitive disorders [18,19,20,21].

However, the prevalence of neurodegenerative disorders is increasing overall, partly because of longer life spans. Unfortunately, today there is no cure for either of these diseases, despite ongoing research efforts [22,23]. Neurodegenerative diseases represent an continuing public health challenge; identifying effective preventive measures and disease-modifying treatments is a current necessity [24].

## 2. Molecular Mechanisms Involved in the Onset of Neurodegenerative Diseases

Neurodegenerative diseases differ in the accumulation of specific proteins and in the different anatomical areas in which the pathological neuronal variation is observed, but they share many of the pathological mechanisms, such as proteotoxic stress and abnormalities in the resulting catabolic systems, that lead to neuronal dysfunction and death. Indeed, in neurodegenerative diseases, the ubiquitin–proteasome system and the autophagy/lysosomal system are found to be altered. In addition, oxidative stress, apoptotic processes, and neuroinflammation play a crucial role in all neurodegenerative diseases [25]. The first evidence of an ongoing inflammatory phenomenon in AD was described many years ago [26], and several subsequent studies have also documented an active inflammatory process in PD, amyotrophic lateral sclerosis (ALS), multiple sclerosis (MS), and a growing number of other nervous system diseases. In these disorders, inflammation is not the trigger for the disease process, but rather, as revealed by studies using animal models, an inflammatory response involving the microglia and astrocytes, which may contribute to the process of disease progression. Therefore, the hypothesis that inhibition of the inflammatory response may be a viable strategy to reverse or slow the course of the disease is well-founded. In the brain, the immune response is performed mainly by the microglia, which influence neurons, astrocytes, and glial cells that perform a supporting function. Microglia typically only become activated to produce inflammatory and neurotrophic factors in order to repair tissue damage induced by an injury or microorganism once the immune system has been activated under pathological conditions. However, a sustained inflammatory state shows the persistence of the inflammatory stimulus and the failure to resolve the damage. Thus, the inflammatory response that physiologically represents the initiation of a beneficial process, i.e., for tissues to ensure the removal of cellular debris, can, under certain conditions, become a risk factor. Prolonged and uncontrolled inflammation can lead to the production of neurotoxic factors capable of inducing significant tissue pathology.

The expression of genes involved in amplifying inflammatory responses results when cells perceive evidence of infection or injury. Inflammation that occurs in response to infectious agents usually begins because of the activation of recognition receptors such as toll-like receptors (TLRs), which recognize certain pathogen-associated molecules that are not present in the host. The cells most involved in the innate immune response, such as macrophages and microglia, highly express these receptors. In recent years, the hypothesis that these recognition receptors may also respond to endogenous molecules, such as those released during necrotic processes, has been confirmed [27,28]. Purinergic receptors are also expressed on microglia and astrocytes and come into play in some pathological states [29,30].

As mentioned above, the pathological brain features of AD include extracellular amyloid plaques; in addition, the inflammatory response in AD induces an increase in the number, size, and motor activity of microglia, as well as a morphological alteration, whereby they change from branched (resting) to amoeboid (active). In addition, the microglia surrounding the plaques appear to be positive for activation markers and proinflammatory mediators, including MHC class II, COX-2, TNF-α, and some interleukins [31,32,33,34]. Proinflammatory cytokines, such as TNF-α, IL-1β, and IL-6, can act directly on the neurons to induce apoptosis [35,36], and the activation of caspases and signal-dependent transcription factors, such as NF-κB and AP-1, leads to the production of many amplifiers (e.g., IL-1β, TNF-α, IL-6). Finally, communication between the neurons and the glia can amplify the production of neurotoxic factors that contribute to AD pathology; molecules with a pro-inflammatory effect on the neurons can increase Aβ production and microglia-mediated inflammation [37].

The inflammatory reaction, characterized by a discharge of chemokine and ROS, is centrally triggered in several acute brain diseases, including ischemic stroke, leading to neuroinflammation, which is often followed by necrosis and apoptosis.

Immune mediators play a key proinflammatory role, enhancing brain cells and promoting the penetration of inflammatory cells such as macrophages, T cells, and neutrophils into the area affected by the ischemic insult. Cytokines such as IL-6 and TNF-α appear to be crucial mediators of post-ischemic inflammation [38].

## 3. Limitations of Current Therapies Used for Treating Neuroinflammation and Neurodegenerative Disorders in the Early Stages of the Disease

Numerous risk factors and diseases can be considered as predisposing factors for neurodegenerative diseases, including cerebrovascular disease, hyperlipidemia, smoking, diabetes, obesity, and traumatic brain injury. In contrast, protective factors are less numerous and include increased cognitive reserve, consumption of a Mediterranean diet, and regular exercise [6,39,40]; however, to date, there are no specific completely effective therapies [41]. Currently, pharmacological treatments have been proven to change the course of AD, although therapies targeting aspects of both amyloid and tau protein are under active investigation. Thus, the clinical management of AD aims to improve patient symptoms and optimize quality of life. Acetylcholine (ACh), a widely distributed neurotransmitter in the body known to improve cognition, is reduced in patients with AD; in fact, the increase in the ACh level induced by the use of acetylcholinesterase inhibitors (e.g., donepezil, rivastigmine, and galantamine) has been associated with improved cognitive ability [42,43,44,45]. Memantine, an n-methyl-d-aspartate (NMDA) receptor antagonist capable of acting by suppressing glutamate-mediated excitotoxicity, has been shown to reduce clinical deterioration in patients with moderate-to-severe AD compared with the controls [46,47,48], but this is not the case in patients with mild disease [49]. Combining acetylcholinesterase inhibitors and memantine may have marginal benefits over single-drug treatment [50]. In addition, it is necessary to consider the modest benefits of these treatments and the potential side effects of each treatment option. Even in the current treatment of Parkinson’s disease, available drug therapies do not represent a complete resolution of symptoms; while they often offer reasonable control of motor symptoms [51,52], they do not alter the evolution of the disease [53]. Drug therapies targeting the motor features of Parkinson’s disease act by potentiating dopamine signaling, involving the direct implementation of the neurotransmitter dopamine (e.g., levodopa), monoamine oxidase (MAO) enzyme inhibition (e.g., selegiline), and catechol-o-methyltransferase (COMT) inhibition (e.g., entacapone). Anticholinergics (e.g., trihexyphenidyl and benztropine) are effective in patients with a predominant tremor phenotype, and their use is preferred in the early stages of Parkinson’s disease. Deep brain stimulation (DBS) can relieve motor fluctuations and tardive dyskinesias in patients with drug-refractory PD, providing benefits for tremor and rigidity, but gait, balance, and cognitive abilities are unlikely to improve as a result of this treatment [54]. Electrodes placed in the inner globe pallidum or subthalamic nucleus regulate abnormal neural impulses, thereby alleviating motor symptoms [55,56,57,58]. Co-treatment with DBS may reduce the dose or adverse effects of medications, but the possibility of some complications, such as bleeding and infection, should be considered here [59,60,61]. The psychiatric manifestations of AD sometimes respond to symptomatic treatments for AD but often require more specific treatment with psychiatric medications. Depression, for example, is often treated with selective serotonin reuptake inhibitors (SSRIs) with low anticholinergic properties (e.g., citalopram, escitalopram, and fluoxetine) [62]. Treatment with neuroleptic drugs should be avoided because they are largely ineffective, and at the same time, risky because of the resulting side effects and increased risk of mortality; however, when nonpharmacological treatments yield insufficient results, the use of these drugs becomes necessary [63]. In DLB and PD, dopaminergic therapy is used to counteract extrapyramidal symptoms, even though this method provides reduced symptomatic improvement than that observed in PD. Depression and anxiety in DLB and PD can be treated with SSRIs or serotonin-norepinephrine reuptake inhibitors (SNRIs). Atypical antipsychotics may be useful for psychiatric symptoms, but should be used with great caution due to their adverse effects on movement and cognition. Traditional neuroleptics should be avoided because of neuroleptic hypersensitivity in patients with DLB. Disease-modifying agents are not yet clinically available [64]. Furthermore, treatment with dopamine agonists can cause side effects in the Parkinsonian patient, which also include changes in behavior, probably because of overstimulation of the mesolimbic dopamine receptors, in predisposed subjects, and this may force the interruption of dopamine (DA) therapy or its substitution with alternate drugs [65].

## 4. The Potential Role of *Andrographis paniculata* in Treating Neuroinflammation and Neurodegenerative Diseases

**Figure 1:**
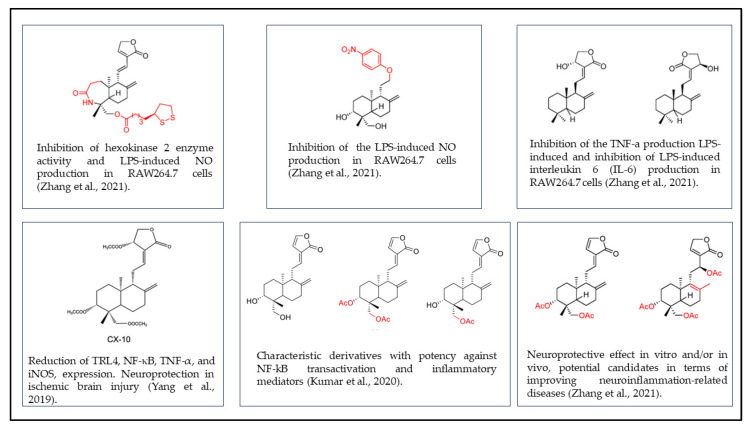
Chemical structures of Andro derivatives with anti-inflammatory and neuroprotective activity. Readapted by: Zhang et al., 2021 [71]; Kumar et al., 2020 [72]; Yang et al., 2019 [73].

Despite research efforts, treatments currently used for neurodegenerative diseases, as previously noted, only provide marginal symptomatic benefits. In recent years, research has focused on products of natural origin, such as phytocompounds, that could be valuable tools in combating various diseases [66,67,68,69,70]. Among the most studied medicinal plants, *Andrographis paniculata* (Ap) (Burm.F.) Wall. ex Nees is known for its active phytochemical content. It is mainly constituted of high amount of alkaloids (63.75%), and flavonoids (23.01%), a moderate amount of tannins (less than 5%), and traces of phenols, saponins, glycosides, steroids, terpenoids, anthocyanins, and carotenoids. These phytochemicals can act individually or in synergy to produce an antiviral, antimicrobial, antitumor, anti-inflammatory, and analgesic effect [71,72]. The analysis of phytochemicals contained in Ap extracts, performed by Koteswara et al., reported that in addition to the previously known compounds (e.g., andrographolide, β-sitosterol, cinnamic acid, caffeic acid, ferulic acid, chlorogenic acid, etc.; and 14-deoxy-11, 12-didehydroandrographolide, neoandrographolide, andrographoside, and 14-deoxyandrographolide 7-methyldihydroogonin, etc.), they also discovered two new flavonoids: (2S)-5,7,20,30-tetramethoxyflavanone and 5-hydroxy-7,20,30-trimethoxyflavanone, characterizing them using 2D NMR spectroscopy to highlight their antioxidant activity. Andrographolide (Andro) is one of the main constituents extracted from Ap leaves. Andro, a diterpene lactone, has attracted particular interest [73,74,75,76,77]. Ap is a plant of Asian origin, known since ancient times by the name “King of Bitters”. Ap, an annual shrub in the family Acanthaceae, is used in traditional Chinese medicine as an antidote for poisonous insect stings and snake bites and to treat dyspepsia, flu, and dysentery. In preclinical studies, the aqueous extract of Ap was able to reduce the expression of inflammatory markers such as TNF-α, IL-1β, IL-6 and oxidative stress makers such as ROS and thiobarbituric acid reactive substances (TBARS) with an enhancement of SOD (superoxide dismutase), CAT (catalase), and GSH (glutathione) activity [78]. In addition, pharmacokinetic analyses conducted on Wistar rats treated with Ap and Andro extract showed that the compounds could easily cross the blood–brain barrier and generate effects at the neuronal level because of their capacity for high distribution in the brain [79]. Especially in the hippocampus, Ap can reduce the activity of LPS-induced cholinesterases, thus exhibiting anti-inflammatory activity and improving mnemonic processes, as confirmed by spatial learning tests [78]. The role of Andro and its derivatives has also been studied in the context of neuroinflammation. Kumar et al., in 2020, and Zhang et al., in 2021, published two review articles concerning Andro derivatives, focusing on alterations in the different functional groups in regards to changes in biological activity. By carefully reviewing the literature from recent years and individually examining the studies performed on each derivative, they reported a collection of the chemical structures of all the derivatives, defining for each the specific biological activity observed in the various in vivo and in vitro study models [80,81]. Figure 1 summarizes the derivatives that can improve inflammation and neuroinflammation by reporting their structure formulas.

A study performed in vivo by LPS-induced neuroinflammation showed that treatment with Andro reduced cortical levels of certain chemokines, such as CCL2 and CCL5. The same authors observed the anti-inflammatory effect of Andro in vitro by inducing the neuroinflammation of astrocytes using both LPS and IL-1β. In the LPS-induced neuroinflammation model, Andro treatment was able to inhibit the LPS-induced TNF-α release through the inhibition of NFkB and JNK, additionally increasing the nuclear factor erythroid 2–related factor 2 (NRF2) levels and heme oxygenase-1 (HO-1) expression through the p38MAPK and ERK-dependent signaling pathway [82]. Additionally, in the IL-1β-induced neuroinflammation model, the compound was able to reduce the release of CCL5, phosphorylation of NFkB p65 and IkBα, and glial fibrillary acidic protein (GFAP) [83]. To investigate the role of Andro in LPS-induced neuroinflammation and memory, Das et al. evaluated the expression of TLR4 and its endogenous ligand high mobility group box 1 (HMGB1) in primary mixed glial cells (PMLCs) in adult prefrontal cortices. Andro treatment was successful in reducing TLR4 expression and LPS-induced *p*-NFκB-p65 activation. In the microglia, however, a reduction in CD-68 expression levels and an increase in arginase-1 levels were observed. However, at the cortical level, Andro was able to reduce the expression of GFAP, exerting a neuroprotective action in the astrocytes through the inhibition of the release of pro-inflammatory molecules such as iNOS, COX-2, nitrates, NLRP3, and caspase-1. Finally, in cognitive memory processes, Andro antagonized the overexpression of PKC and the phosphorylation of cAMP-responsive element binding protein (CREB), which usually results in upregulated cognitive deficits. The same authors also observed that at the level of the prefrontal cortex, Andro reduced the levels of β-amyloid, APP, ptau, BACE-1 (β-secretase-1), as well as the activation of the pro-apoptotic caspase-3 and bax genes, and increased the levels of the anti-apoptotic gene Bcl-2, improving neuronal viability. At the hippocampal level, an increase in postsynaptic density protein 95 (PSD-95) and synapsin was observed, resulting in increased neuronal plasticity, reducing the expression levels of inflammatory cytokines such as TNF-α and macrophage inflammatory protein-1 (MIP-1), and an increasing those of TGF-β and IL-10 [84]. In another study conducted by Adedayo et al., the effect of Andro in counteracting the amnesic effect of scopolamine, like that found in AD, was evaluated. Rats treated with the aqueous extract of Ap showed an improvement in cognitive function, as assessed by the Y-Labyrinth Test and the Morris Water Maze (MWM) Test, and additional analyses showed significant reductions in AChE and Buttiril-ChE, as well as a reduction in some MAOs and purinergic enzymes such as ATPdase and ADPdase, while the levels of 5-nucleotidase and adenosine deaminase were found to be increased at the hippocampal level [85].

Because neuroinflammation also plays a crucial role in diseases such as depression and schizophrenia [86,87], some studies aimed to investigate the possible role of Andro in these diseases. In an in vivo model of schizophrenia induced by the administration of phencyclidine (PCP), Andro reduced the levels of IL-1β and TNF-α, *p*-p65, *p*-IκBα, *p*-p38, and *p*-ERK1/2 in the prefrontal cortex; improved oxidative stress through the activation of antioxidant enzymes such as SOD, CAT, and GSH-Px; and increased the levels of NRF-2, HO-1, and NAD(P)H Quinone Dehydrogenase 1 (NQO-1). Cognitive improvement was also confirmed by behavioral tests that showed increased locomotor activity [88]. Andro was also found to be effective in the treatment of stress-induced depression. Mice subjected to chronic unpredictable mild stress (CUMS) and treated with Andro showed reduced levels of NO, iNOS, IL-1β, IL-6, TNF-α, COX-2, *p*-p65, *p*-IκBα, and the NLR family pyrin domain containing 3 (NLRP3) in the prefrontal cortex, compared with the levels in untreated mice. In addition, Andro exerted a pro-autophagic action, through increased Beclin-1 expression, and decreased *p*-mTOR [89].

A recent study has evaluated Andro’s effects in the treatment of neurological disorders induced by heavy metal exposure. Specifically, the effects of Andro on aluminum intoxication in Drosophila were assessed. Interestingly, Andro treatment on flies improved survival, locomotor performance, and learning and memory through reduced AChE and MAO activity and increased catalase activity [90].

In 2005, Iruretagoyena et al. also observed the protective action of Andro in the treatment of Multiple Sclerosis, an autoimmune neurodegenerative disease in which immune defenses, particularly lymphocytes, attack nervous system components. Indeed, in dendritic cells (DCs) pulsed with hen’s egg ovalbumin (OVA), Andro counteracted the generation of peptide-MHC complexes required for T-cell activation by inhibiting the upregulation of maturation markers I-Ab, CD40, and CD86 in LPS-treated dendritic cells. Andro treatment was also tested in vivo on C57BL/6 mice with experimental autoimmune encephalomyelitis (induced with the peptide MOG35-55), and it significantly reduced the incidence of the disease, as also shown by the reduced production of IFN and IL-2 [91]. Therefore, based on the data reported in the literature, we can state that both Ap and Andro are effective in treating the inflammatory and degenerative effects that occur in the nervous system during various disease states [75,78,92,93] (Table 1). Here, we will examine these effects on diseases involving the nervous system, focusing on those most prevalent in the world population.

### 4.1. Alzheimer Disease

In vitro studies showed that Andro was able to activate α-secretase, which is involved in the inhibition of Aβ formation, and to inhibit β-secretase, which is involved in Aβ formation, without the protective treatment causing toxicity [94]. On hippocampal neuronal cells of HT-22 mice, treatment with Andro increased the levels of nuclear factor erythroid-derived 2-like 2/Kelch-like ECH-associated protein 1 (NRF2/Keap), the antioxidant response element (ARE) gene, and the HO-1 enzyme. Since these factors are major players in anti-inflammatory and antioxidant responses, it is evident that Andro induced a cytoprotective response in the brain [95]. On mouse microglia BV-2 (microglial cells derived from C57BL/6 murine), Andro reduced the expression of Aβ, improving neuronal viability, and through the NFkB-mediated signaling pathway, reduced the levels of TNF-α, COX-2, and PGE2, i-NOS, NO, and cytokines such as IL-1β, IL-6, protecting the neurons from damage produced by inflammation [96].

In addition to Andro, its analogs have also been used on these same cells, achieving complementary results. Indeed, the analogs have shown the ability to inhibit LPS-induced NO production and iNOS expression, as well as TNF-α and IL-6 production [97].

One enzyme known to be involved in learning and memory processes, and especially in tau protein phosphorylation and increased β-amyloid production, is glycogen synthase kinase-3β (GSK3β) [98]. Andro treatment of primary hippocampal neuronal cultures induced the inhibition of the GSK3β enzyme and the reduction of its active form [99]. The same treatment conducted on human embryonic kidney (HEK293) cells showed comparable results. Because GSK3β is involved in the Wnt/β-catenin signaling pathway, known for its role in neurogenesis, the effect of Andro on this pathway was also investigated [100]. Andro treatment on hippocampal cells showed an induction of Wnt gene transcription by restoring its proper activity [99]. Several studies regarding the effects of Ap have also been conducted in mouse models. Various transgenic mouse models have been used in the literature to reproduce the features of Alzheimer’s disease [101,102].

In one study in which Andro was tested on 2-month-old mice exhibiting mutations on the genes encoding for APP and presenilin (PS1), known proteins involved in AD, increased expression of the Wnt/β-catenin signaling pathway was observed [103]. In another experiment, in which 7–12-month-old mice were used, Andro was able to alter the maturation of amyloid plaques in the cortex and the hippocampus; in the early stages of the disease, the number of plaques was found to be reduced. Andro was also observed to exert its effects on tau protein phosphorylation, leading to a significant increase in post-synaptic proteins, including Shank, GluN2B, GluA2, PSD-95, as well as an increase in the inactive form of GSK3β. Moreover, behavioral tests found that Andro-treated mice showed improved learning and lower latency values, as well as improved spatial memory performance [104]. In addition to the transgenic mouse models, some work has also been conducted on Octodon degus mice, which can naturally reproduce the neurodegeneration and neurological signs of Alzheimer’s with advancing age [102]. Rivera et al. performed several behavioral tests on these 12- and 56-month-old rodents to assess their degree of spatial learning and memory after Andro administration. The results obtained showed neuroprotective effects that resulted in the recovery of memory and learning, enhancement of the excitatory postsynaptic field potential (fEPSP), protection of specific proteins, such as synaptophysin (SYP), along with increased vesicular glutamate transporter 1 (vGluT1) and NMDA receptor subunit GluN2A. Their results also showed decreased phosphorylated tau protein and Aβ aggregate maturation in aged mice [105]. In contrast, on these same animals, Lindsay et al. reported the neuroprotective effects of Andro through a reduction in Aβ, GFAP, IL-6, COX-2, and oxidative stress markers such as 4-Hydroxynonenal (4-HNE) () and n-Tyrosin (n-Tyr) in the brain [106].

It is well known that impaired glucose metabolism at the neuronal level may be related to several neurodegenerative diseases, including AD [107,108,109,110,111,112]. Cognitive deficits and symptoms comparable to AD are also reproducible following the intracerebroventricular administration of streptozotocin (SZT).

**Figure 2:**
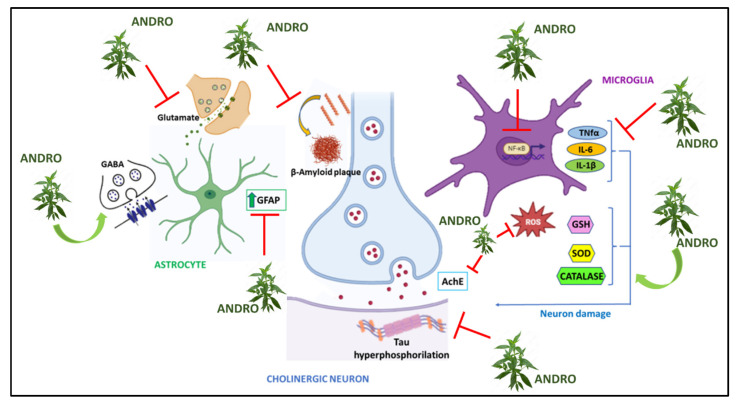
Graphic representation of molecular mechanisms underlying Alzheimer’s Disease on which *Andrographis paniculata* acts.

Rats that underwent this treatment and were treated with Andro show improved spatial memory according to the results of the Morris Water Maze (Morris Water) and the Elevated Plus Maze tests, compared with the results for the untreated group of animals. Andro also reduced levels of neuroinflammatory markers such as TNF-α, IL-1B, and IL-16, decreasing levels of the neurotransmitter glutamate (GLU), and increasing levels of GABA. Because STZ administration induces increased levels of AChE and ptau, the group of animals that had received Andro treatment showed a significant reduction in the expression levels of AChE and ptau, as well as a reduction in oxidative stress through the attenuation of MDA (malondialdehyde) and nitrite, along with increased levels of GSH, SOD, and catalase compared with the levels in the group treated with STZ alone [113]. Comparable results were also obtained for diabetic rats given oral administrations of STZ and subsequently treated with Andro [114]. In another study on rat primary hippocampal neurons, treatment with Andro resulted in increased glucose uptake through increased GLUT translocation and increased ATP production, promoting AMPK-dependent glycolysis [115] (Figure 2).

### 4.2. Parkinson’s Disease

As already widely described, inflammation and apoptosis are essential factors in various neurodegenerative diseases, of which Parkinson’s is one [116,117]. To study the effects of Andro on this disease, rat midbrain glia cultures were pre- and post-treated concomitantly with LPS-induced dopaminergic neurodegeneration. The results obtained showed that Andro was able to attenuate LPS-induced dopaminergic neurodegeneration by reducing the activation of microglia and inflammatory factors such as ROS, TNF-α, NO, and PGE2. In addition, pretreatment with Andro on BV2 microglia cells reduced the expression of COX-2 and iNOS. However, in this work, the neuroprotection of Andro on neurodegeneration induced by 1-methyl-4-phenyl-pyridine (MPP), a metabolite of MPTP used to generate Parkinson’s, was also tested, although pretreatment with Andro failed to reduce neuronal damage [118]. In another study conducted on mice subjected to intraperitoneal administration of MPTP, treatment with Andro produced positive results for all behavioral tests performed, including the catalepsy, grip strength, and, rotarod tests, leading to improved motor conditions [89]. In vitro, it was seen that LPS- and MPP-induced microglia activation was reduced following Andro administration, resulting in decreased NLRP3 inflammasome activation, and these data were confirmed by inhibition of the microglial expression marker Iba-1, both in vitro and in vivo, in the mouse brain [119,120]. Andro has also been shown to improve mitochondrial dysfunction through the inhibition of ROS formation and maintenance of mitochondrial membrane potential (in vitro) by additionally promoting autophagosome formation and the elimination of damaged mitochondria [120]. The neuroprotective effects of Andro on MPP-induced damage were also studied on neuroblastoma cells (SH-SY5Y). In these cells, andrographolide-lipoic acid (AL-1) conjugate protected against MPP-induced damage, leading to increased cell viability and the inhibited phosphorylation of NF-κB p65 and IκBα. Moreover, in MPTP-treated mice, AL-1 protected against the loss of TH-positive dopaminergic neurons in the substantia nigra pars compacta, attenuated dopamine loss in the striatum, and improved motor functions, as showed by behavioral tests [121].

However, in a later study performed in SH-SY5H cells, Ketterman et al. observed that the administration of Andro did not provide protective effects against oxidative stress [119].

**Figure 3:**
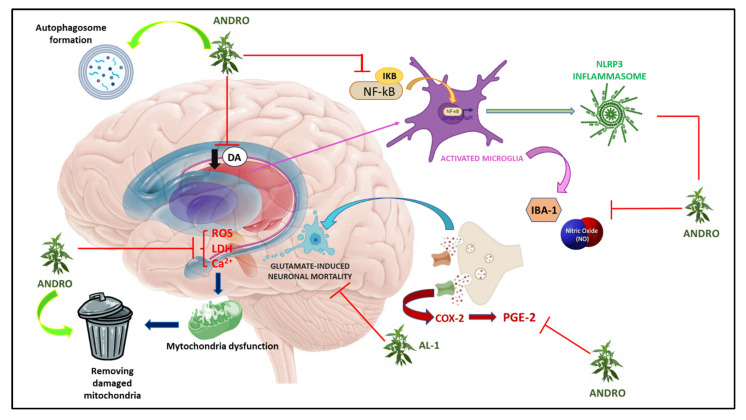
Graphic representation of molecular mechanisms underlying Parkinson’s disease on which *Andrographis paniculata* acts.

Dysregulation of GLU levels is involved in several processes that may cause neuronal damage [122,123,124]. In this regard, in 2014, Yang et al. studied the neuroprotective effect of Ap extract on HT22 neuronal cells in the mouse hippocampus following glutamate-induced damage. The results showed that Ap can significantly reduce GLU-induced neuronal mortality and cytosolic lactate dehydrogenase (LDH) levels, Ca^2+^ influx, and intracellular ROS production induced by GLU in a dose-dependent manner. In addition, the same authors also observed a significant decrease in the phosphorylation of MAPK, p38, ERK, and JNK. At the same time, Western blotting analysis revealed a restoration of the levels of anti-apoptotic proteins such as Bcl-2, Bid, and Bax, and a reduction in apoptosis-inducing factor (AIF) after Andro treatment [95] (Figure 3).

### 4.3. Brain Ischemia-Reperfusion Injury

Cerebral ischemia is a pathological condition characterized by a reduced blood supply that can affect different brain areas and induce significant neuronal damage [125]. In a study conducted on mouse brain endothelial cells, no positive effects were found following the administration of Andro, as the compound not only failed to improve the viability of these cells, but also induced a marked reduction in the number of neuronal cells due to increased LDH release and increased apoptosis, blocking cell growth in the G0/G1 phase [126]. However, from many other studies, especially those conduced in recent years, the protective action of Andro on ischemic damage seems to emerge and to be confirmed. More specifically, in a rat–mouse model with permanent middle cerebral artery occlusion (pMCAO), Andro reduced the infarct volume and microglia activation in peri-infarct areas by combatting increased levels of inflammatory markers such as IL-1β, TNF-α, and PGE2 and the transcription factor NFkB [127]. In another study, however, rats undergoing ischemic brain injury/reperfusion (CI/R) treated with Andro showed decreased cerebral infarction, reduced superoxide anion and nitrotyrosine, reduced expression of gp91phox/NOX2, IL-1β and hypoxia inducible factor (HIF), as well as reduced NFkB p65, thus confirming the protective effects of Andro in counteracting an ischemic attack [73]. Mice with CI/R-induced oxidative brain damage and treated with Andro also reported a reduced magnitude of infarction and subsequent neurological deficits, accompanied by reduced free radical production, as well as reduced nitrotyrosine, CD11b, NOX2, and iNOS formation [128].

**Figure 4:**
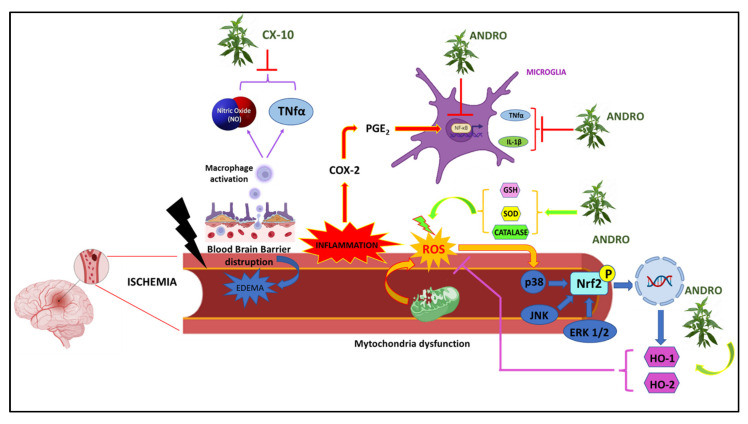
Graphic representation of molecular mechanisms underlying Ischemia on which *Andrographis paniculata* acts.

Another andrographolide derivative, triacetylandrographolide (CX-10), has been used to test for possible neuroprotective effects against cerebral ischemia. On the macrophage cell line RAW264.7, both Andro and CX-10 counteracted NO and TNF-α production, while in BALB rats, CX-10 reduced LPS-induced TNF-α production. In Sprague Dawley rats with middle cerebral artery occlusion (MCAO), reduced infarct size and improved motor performance were observed. At the level of brain tissues, CX-10 reduced the levels of TNF-α and IL-1β and increased the activity of antioxidant enzymes such as SOD, CAT, and GSH-P. Western blot analysis also showed the positive effects of Andro in reducing the expression levels of TLR4, NF-κB, TNF-α, and iNOS proteins by increasing the expression of nuclear factor erythroid 2-related factor 2 (NRF2) and HO-1 [128]. In mouse cerebral endothelial cells (CECs) treated with 10μM ANDRO, an increase in HO-1 expression mediated by the phosphorylation of ERK1/2, p38 MAPK, and JNK1/2, and an increase was observed in Heme oxygenase 2 (HO-2) protein, via the NRF2 signaling pathway. In this way, Andro protected neuronal cells from cell death induced by oxygen-glucose deprivation (OGD), thus improving cell viability, while in rats subjected to MCAO, Andro’s antioxidant effect in counteracting free radical production was observed, and a reduction in cerebral edema and infarct volume was detected in brain tissue analyses [129] (Figure 4).

## 5. Conclusions

In conclusion, Ap and Andro exert neuroprotective effects on different NDs. The emerging information on the cytoprotective, anti-inflammatory, and antioxidant effects caused by the properties in Ap and Andro seems to explain many mechanisms at the heart of neuroprotective action. In particular, Andro can exert protective and therapeutic effects on certain central nervous system disorders, such as AD, PD, and cerebral ischemia, through its antioxidant and anti-inflammatory mechanisms. The neuroprotective effects of Andro may be mediated by several mechanisms, such as restoring blood–brain integrity, increasing synaptic plasticity, reducing the levels of specific chemokines and pro-inflammatory molecules activated during the neuroinflammation processes, promoting apoptosis and neurogenesis, and inhibiting Aβ aggregation. Indeed, both in vivo and in vitro studies have suggested that Andro shows considerable potential for development as a novel drug in the treatment of neurodegenerative diseases, and for this reason, it could also be considered as an adjuvant to conventional drugs therapies. Thus, a greater understanding of the neuroprotective molecular mechanisms of Ap and Andro could be crucial for identifying new therapeutic targets in NDs. However, in addition to its neuroprotective role, Ap and its major constituent Andro exhibit multiple pharmacological activities, including antioxidant, anti-inflammatory, anticancer, antimicrobial, parasitic, hepatoprotective, antihyperglycemic, and antihyperglycemic actions, and experimental evidence has suggested that Andro may antagonize uncomplicated upper respiratory tract infections, including sinusitis and the common cold, as well as exert effects combatting rheumatoid arthritis. Furthermore, Andro does not appear to be particularly toxic; in fact, several studies have evaluated its efficacy and toxicity, even at high doses, demonstrating that it is a well-tolerated compound. However, some side effects have been documented, be problematic regarding the use of Andro, including gastrointestinal, skin, and subcutaneous tissue disorders, as well as anaphylaxis, general disorders and abnormal administration site conditions, and system disorders. Most of these adverse effects are mild to moderate, while a small number of patients may experience serious or life-threatening adverse effects. Most of these adverse reactions caused by Ap derivative injections can be relieved after drug discontinuation and symptomatic treatment [130]. Therefore, despite the fact that the use of Ap and Andro shows clear advantages, it is still necessary to carry out additional studies to improve the understanding of its therapeutic mechanisms and above all, to better understand and limit the disadvantages linked to its administration.

## Figures and Tables

**Table 1:** Effects of Ap and Andro on various animal and cellular models and on various tissues.

Target	Effects	Refs.
**Effects on proinflammatory** **molecules and on expression of oxidative stress mediators**	Reduction of TNF-α, IL-1β, IL-6, ROS, and TBARS expression	Sani et al., 2019 [78]
	Reduction of chemokine ligand 5 (CCL5) release, phosphorylation of NFkB p65 and IkBa, as well as GFAP (glial fibrillary acidic protein) induced by IL-1b	Wong et al., 2014 [83] Wong et al., 2016 [82]
	LPS-induced reduction of TLR4 expression and *p*-NFκB-p65 activation; reduction in the levels of inflammatory cytokines, such as TNF-α and MIP-1 (macrophage inflammatory protein-1); increase in anti-inflammatory cytokines, such as TGF-β and IL-10; reduction of pro-inflammatory molecule expression, such as iNOS, COX-2, nitrates, NLRP3, caspase-1	Das et al., 2017 [84]
	Improvement of SOD (superoxide dismutase), CAT (catalase), and GSH (glutathione) activity	Sani et al., 2019 [78]
**Effects on the hippocampus**	Reduction of cholinesterase activity induced by lipopolysaccharide, showing anti-inflammatory activity and improving memory	Sani et al., 2019 [78]
	Increased expression of PSD-95 (postsynaptic density protein 95) and synapsin, which are involved in synaptic plasticity	Das et al., 2017 [84]
	Increase in purinergic enzymes, such as ATPdase, ADPdase, 5-nucletidase, and adenosine deaminase	Adedayo et al., 2021 [85]
**Effects on models of** **neuroinflammation**	Reduction of cortical levels of chemokines, such as CCL2, CCL5	Wong et al., 2016 [82]
**Effects on astrocytes**	Increased NRF2 levels and HO-1 expression through the p38 MAPK and ERK dependent pathway; anti-inflammatory effect in vitro; reduction of GFAP expression	Das et al., 2017 [84]
**Effects on microglia**	Reduction of CD-68 expression; increased levels of arginase-1	Das et al., 2017 [84]
**Effects on prefrontal** **cortex**	Reduction in β-amyloid, APP, ptau, BACE-1 (β-secretase-1) levels; activation of caspase-3 and bax; increased levels of the anti-apoptotic gene Bcl-2	Das et al., 2017 [84]
**Effects on the activity of neurotransmitters**	Reduction of AChE and Buttyryl-ChE, and of monoamine oxidases (MAO).	Adedayo et al., 2021 [85]
**Effects on schizophrenia**	Reduction of IL-1β and TNF-α, *p*-p65, *p*-IκBα, *p*-p38, and *p*-ERK1/2 levels in the prefrontal cortex; activation of antioxidant enzymes such as SOD, CAT, and GSH-Px; increased levels of NRF-2, HO-1, and NQO-1; increased locomotor activity [88]	Wang et al., 2021 [88]
**Effects on stress-induced depression**	Reduction of NO, iNOS, IL-1β, IL-6, TNF-α, COX-2, *p*-p65, *p*-IκBα levels and NLRP3 inflammasome assembly in the prefrontal cortex; pro-autophagic action through an increase in Beclin-1 expression and a reduction in *p*-mTOR	Geng et al., 2019 [89]
**Effects in aluminum poisoning**	Improved survival, locomotor performance, learning, and memory through a reduction of AChE and MAO activity and increased catalase activity	Adedayo et al., 2021 [85]
**Effects on Multiple** **Sclerosis**	Prevention of the generation of peptide-MHC complexes required for T cell activation; inhibition of upregulation of maturation markers I-Ab, CD40, and CD86 in LPS-treated dendritic cells	Adedayo et al., 2021 [85]
**Effects on experimental autoimmune encephalomyelitis**	Significant reduction in incidencem of disease, as demonstrated by the reduced production of IFN and IL-2	Adedayo et al., 2021 [85]

## Data Availability

Not applicable.

## References

[1] Prusiner S.B. (2013). Biology and genetics of prions causing neurodegeneration. Annu. Rev. Genet..

[2] Erkkinen M.G., Kim M.O., Geschwind M.D. (2018). Clinical Neurology and Epidemiology of the Major Neurodegenerative Diseases. Cold Spring Harb. Perspect. Biol..

[3] Gonzales M.M., Garbarino V.R., Pollet E., Palavicini J.P., Kellogg D.L., Kraig E., Orr M.E. (2022). Biological aging processes underlying cognitive decline and neurodegenerative disease. J. Clin. Investig..

[4] Walker D.G., Lue L.F., Serrano G., Adler C.H., Caviness J.N., Sue L.I., Beach T.G. (2015). Altered Expression Patterns of Inflammation-Associated and Trophic Molecules in Substantia Nigra and Striatum Brain Samples from Parkinson’s Disease, Incidental Lewy Body Disease and Normal Control Cases. Front. Neurosci..

[5] Reitz C., Rogaeva E., Foroud T., Farrer L.A. (2011). Genetics and genomics of late-onset Alzheimer’s disease and its endophenotypes. Int. J. Alzheimers Dis..

[6] Mayeux R., Stern Y. (2012). Epidemiology of Alzheimer disease. Cold Spring Harb. Perspect. Med..

[7] Sosa-Ortiz A.L., Acosta-Castillo I., Prince M.J. (2012). Epidemiology of dementias and Alzheimer’s disease. Arch. Med. Res..

[8] Porsteinsson A.P., Isaacson R.S., Knox S., Sabbagh M.N., Rubino I. (2021). Diagnosis of Early Alzheimer’s Disease: Clinical Practice in 2021. J. Prev. Alzheimers Dis..

[9] Garre-Olmo J. (2018). Epidemiology of Alzheimer’s disease and other dementias. Rev. Neurol..

[10] Rostagno A.A. (2022). Pathogenesis of Alzheimer’s Disease. Int. J. Mol. Sci..

[11] Niu H., Álvarez-Álvarez I., Guillén-Grima F., Aguinaga-Ontoso I. (2017). Prevalence and incidence of Alzheimer’s disease in Europe: A meta-analysis. Neurologia.

[12] Zhang X.X., Tian Y., Wang Z.T., Ma Y.H., Tan L., Yu J.T. (2021). The Epidemiology of Alzheimer’s Disease Modifiable Risk Factors and Prevention. J. Prev. Alzheimers Dis..

[13] Balestrino R., Schapira A.H.V. (2020). Parkinson disease. Eur. J. Neurol..

[14] Moisan F., Kab S., Mohamed F., Canonico M., Le Guern M., Quintin C., Carcaillon L., Nicolau J., Duport N., Singh-Manoux A. (2016). Parkinson disease male-to-female ratios increase with age: French nationwide study and meta-analysis. J. Neurol. Neurosurg. Psychiatry.

[15] Raket L.L., Oudin Åström D., Norlin J.M., Kellerborg K., Martinez-Martin P., Odin P. (2022). Impact of age at onset on symptom profiles, treatment characteristics and health-related quality of life in Parkinson’s disease. Sci. Rep..

[16] Lippa C.F., Knopman D.S. (2007). Dementia: Many roads, but not built in a day. Neurology.

[17] Jellinger K.A. (2018). Dementia with Lewy bodies and Parkinson’s disease-dementia: Current concepts and controversies. J. Neural. Transm..

[18] Thenganatt M.A., Jankovic J. (2014). Parkinson disease subtypes. JAMA Neurol..

[19] Alves G., Larsen J.P., Emre M., Wentzel-Larsen T., Aarsland D. (2006). Changes in motor subtype and risk for incident dementia in Parkinson’s disease. Mov. Disord..

[20] Fang C., Lv L., Mao S., Dong H., Liu B. (2020). Cognition Deficits in Parkinson’s Disease: Mechanisms and Treatment. Park. Dis..

[21] Aarsland D., Batzu L., Halliday G.M., Geurtsen G.J., Ballard C., Ray Chaudhuri K., Weintraub D. (2021). Parkinson disease-associated cognitive impairment. Nat. Rev. Dis. Primers.

[22] Heemels M.T. (2016). Neurodegenerative diseases. Nature.

[23] Lamptey R.N.L., Chaulagain B., Trivedi R., Gothwal A., Layek B., Singh J. (2022). A Review of the Common Neurodegenerative Disorders: Current Therapeutic Approaches and the Potential Role of Nanotherapeutics. Int. J. Mol. Sci..

[24] Logroscino G., Urso D., Savica R. (2022). Descriptive Epidemiology of Neurodegenerative Diseases: What Are the Critical Questions?. Neuroepidemiology.

[25] Dugger B.N., Dickson D.W. (2017). Pathology of Neurodegenerative Diseases. Cold Spring Harb. Perspect. Biol..

[26] Akiyama H. (1994). Alzheimer’s disease and the immune system response. Nihon Rinsho.

[27] Balistreri C.R., Colonna-Romano G., Lio D., Candore G., Caruso C. (2009). TLR4 polymorphisms and ageing: Implications for the pathophysiology of age-related diseases. J. Clin. Immunol..

[28] Liang W., Han B., Hai Y., Liu Y., Liu X., Yang J., Sun D., Yin P. (2022). The Role of Microglia/Macrophages Activation and TLR4/NF-κB/MAPK Pathway in Distraction Spinal Cord Injury-Induced Inflammation. Front. Cell Neurosci..

[29] Di Virgilio F., Ceruti S., Bramanti P., Abbracchio M.P. (2009). Purinergic signalling in inflammation of the central nervous system. Trends Neurosci..

[30] Aminin D., Illes P. (2021). Purinergic Signaling in Neuroinflammation. Int. J. Mol. Sci..

[31] Akiyama H., Barger S., Barnum S., Bradt B., Bauer J., Cole G.M., Cooper N.R., Eikelenboom P., Emmerling M., Fiebich B.L. (2000). Inflammation and Alzheimer’s disease. Neurobiol. Aging.

[32] Cartier L., Hartley O., Dubois-Dauphin M., Krause K.H. (2005). Chemokine receptors in the central nervous system: Role in brain inflammation and neurodegenerative diseases. Brain Res. Brain Res. Rev..

[33] Rahman M.M., Lendel C. (2021). Extracellular protein components of amyloid plaques and their roles in Alzheimer’s disease pathology. Mol. Neurodegener..

[34] Muzio L., Viotti A., Martino G. (2021). Microglia in Neuroinflammation and Neurodegeneration: From Understanding to Therapy. Front. Neurosci..

[35] McCoy M.K., Tansey M.G. (2008). TNF signaling inhibition in the CNS: Implications for normal brain function and neurodegenerative disease. J. Neuroinflammation.

[36] Simi A., Tsakiri N., Wang P., Rothwell N.J. (2007). Interleukin-1 and inflammatory neurodegeneration. Biochem. Soc. Trans..

[37] Glass C.K., Saijo K., Winner B., Marchetto M.C., Gage F.H. (2010). Mechanisms underlying inflammation in neurodegeneration. Cell.

[38] Maida C.D., Norrito R.L., Daidone M., Tuttolomondo A., Pinto A. (2020). Neuroinflammatory Mechanisms in Ischemic Stroke: Focus on Cardioembolic Stroke, Background, and Therapeutic Approaches. Int. J. Mol. Sci..

[39] Petersson S.D., Philippou E. (2016). Mediterranean Diet, Cognitive Function, and Dementia: A Systematic Review of the Evidence. Adv. Nutr..

[40] Hu C.J., Octave J.N. (2019). Editorial: Risk Factors and Outcome Predicating Biomarker of Neurodegenerative Diseases. Front. Neurol..

[41] Durães F., Pinto M., Sousa E. (2018). Old Drugs as New Treatments for Neurodegenerative Diseases. Pharmaceuticals.

[42] Birks J.S., Harvey R. (2018). Donepezil for dementia due to Alzheimer’s disease. Cochrane Database Syst. Rev..

[43] Olin J., Schneider L. (2002). Galantamine for Alzheimer’s disease. Cochrane Database Syst. Rev..

[44] Birks J.S., Chong L.Y., Grimley Evans J. (2015). Rivastigmine for Alzheimer’s disease. Cochrane Database Syst. Rev..

[45] Kandiah N., Pai M.C., Senanarong V., Looi I., Ampil E., Park K.W., Karanam A.K., Christopher S. (2017). Rivastigmine: The advantages of dual inhibition of acetylcholinesterase and butyrylcholinesterase and its role in subcortical vascular dementia and Parkinson’s disease dementia. Clin. Interv. Aging.

[46] Howard R., McShane R., Lindesay J., Ritchie C., Baldwin A., Barber R., Burns A., Dening T., Findlay D., Holmes C. (2012). Donepezil and memantine for moderate-to-severe Alzheimer’s disease. N. Engl. J. Med..

[47] Reisberg B., Doody R., Stöffler A., Schmitt F., Ferris S., Möbius H.J., Group M.S. (2003). Memantine in moderate-to-severe Alzheimer’s disease. N. Engl. J. Med..

[48] Folch J., Busquets O., Ettcheto M., Sánchez-López E., Castro-Torres R.D., Verdaguer E., Garcia M.L., Olloquequi J., Casadesús G., Beas-Zarate C. (2018). Memantine for the Treatment of Dementia: A Review on its Current and Future Applications. J. Alzheimers Dis..

[49] McShane R., Areosa Sastre A., Minakaran N. (2019). Memantine for dementia. Cochrane Database Syst. Rev..

[50] Farrimond L.E., Roberts E., McShane R. (2012). Memantine and cholinesterase inhibitor combination therapy for Alzheimer’s disease: A systematic review. BMJ Open.

[51] Nemade D., Subramanian T., Shivkumar V. (2021). An Update on Medical and Surgical Treatments of Parkinson’s Disease. Aging Dis..

[52] Bosco D., Plastino M., Bosco F., Fava A., Rotondo A. (2011). Daily motor performance after switching levodopa to melevodopa: An open-label on advanced Parkinson’s disease with “delayed-on” and/or “wearing-off”. Minerva Med..

[53] Rascol O., Payoux P., Ory F., Ferreira J.J., Brefel-Courbon C., Montastruc J.L. (2003). Limitations of current Parkinson’s disease therapy. Ann. Neurol..

[54] Fasano A., Daniele A., Albanese A. (2012). Treatment of motor and non-motor features of Parkinson’s disease with deep brain stimulation. Lancet Neurol..

[55] Benabid A.L., Pollak P., Louveau A., Henry S., de Rougemont J. (1987). Combined (thalamotomy and stimulation) stereotactic surgery of the VIM thalamic nucleus for bilateral Parkinson disease. Appl. Neurophysiol..

[56] Siegfried J., Lippitz B. (1994). Bilateral chronic electrostimulation of ventroposterolateral pallidum: A new therapeutic approach for alleviating all parkinsonian symptoms. Neurosurgery.

[57] Follett K.A., Weaver F.M., Stern M., Hur K., Harris C.L., Luo P., Marks W.J., Rothlind J., Sagher O., Moy C. (2010). Pallidal versus subthalamic deep-brain stimulation for Parkinson’s disease. N. Engl. J. Med..

[58] Odekerken V.J., Boel J.A., Schmand B.A., de Haan R.J., Figee M., van den Munckhof P., Schuurman P.R., de Bie R.M., NSTAPS Study Group (2016). GPi vs STN deep brain stimulation for Parkinson disease: Three-year follow-up. Neurology.

[59] Lyons K.E., Pahwa R. (2004). Deep brain stimulation in Parkinson’s disease. Curr. Neurol. Neurosci. Rep..

[60] Guridi J., Rodriguez-Oroz M.C., Alegre M., Obeso J.A. (2012). Hardware complications in deep brain stimulation: Electrode impedance and loss of clinical benefit. Park. Relat. Disord..

[61] Pouratian N., Thakkar S., Kim W., Bronstein J.M. (2012). Deep brain stimulation for the treatment of Parkinson’s disease: Efficacy and safety. Degener. Neurol. Neuromuscul. Dis..

[62] Seitz D.P., Adunuri N., Gill S.S., Gruneir A., Herrmann N., Rochon P. (2011). Antidepressants for agitation and psychosis in dementia. Cochrane Database Syst. Rev..

[63] Sink K.M., Holden K.F., Yaffe K. (2005). Pharmacological treatment of neuropsychiatric symptoms of dementia: A review of the evidence. JAMA.

[64] Paolini Paoletti F., Gaetani L., Parnetti L. (2020). The Challenge of Disease-Modifying Therapies in Parkinson’s Disease: Role of CSF Biomarkers. Biomolecules.

[65] Rotondo A., Bosco D., Plastino M., Consoli A., Bosco F. (2010). Clozapine for medication-related pathological gambling in Parkinson disease. Mov. Disord..

[66] Bosco D., Plastino M., Colica C., Bosco F., Arianna S., Vecchio A., Galati F., Cristiano D., Consoli A., Consoli D. (2012). Opioid antagonist naltrexone for the treatment of pathological gambling in Parkinson disease. Clin. Neuropharmacol..

[67] Sharma K. (2019). Cholinesterase inhibitors as Alzheimer’s therapeutics (Review). Mol. Med. Rep..

[68] Islam M.S., Quispe C., Hossain R., Islam M.T., Al-Harrasi A., Al-Rawahi A., Martorell M., Mamurova A., Seilkhan A., Altybaeva N. (2021). Neuropharmacological Effects of Quercetin: A Literature-Based Review. Front. Pharmacol..

[69] Zhang R., Miao Q.W., Zhu C.X., Zhao Y., Liu L., Yang J., An L. (2015). Sulforaphane ameliorates neurobehavioral deficits and protects the brain from amyloid β deposits and peroxidation in mice with Alzheimer-like lesions. Am. J. Alzheimers Dis. Other Demen.

[70] Maiuolo J., Bosco F., Guarnieri L., Nucera S., Ruga S., Oppedisano F., Tucci L., Muscoli C., Palma E., Giuffrè A.M. (2023). Protective Role of an Extract Waste Product from. Plants.

[71] Malahubban M., Alimon A.R., Sazili A.Q., Fakurazi S., Zakry F.A. (2013). Phytochemical analysis of *Andrographis paniculata* and Orthosiphon stamineus leaf extracts for their antibacterial and antioxidant potential. Trop. Biomed..

[72] Yusuf A.L., Adeyemi K.D., Roselina K., Alimon A.R., Goh Y.M., Samsudin A.A., Sazili A.Q. (2018). Dietary supplementation of different parts of *Andrographis paniculata* affects the fatty acids, lipid oxidation, microbiota, and quality attributes of longissimus muscle in goats. Food Res. Int..

[73] Yang M.Y., Yu Q.L., Huang Y.S., Yang G. (2019). Neuroprotective effects of andrographolide derivative CX-10 in transient focal ischemia in rat: Involvement of Nrf2/AE and TLR/NF-κB signaling. Pharmacol. Res..

[74] Dai Y., Chen S.R., Chai L., Zhao J., Wang Y. (2019). Overview of pharmacological activities of. Crit. Rev. Food Sci. Nutr..

[75] Hossain R., Quispe C., Herrera-Bravo J., Beltrán J.F., Islam M.T., Shaheen S., Cruz-Martins N., Martorell M., Kumar M., Sharifi-Rad J. (2022). Neurobiological Promises of the Bitter Diterpene Lactone Andrographolide. Oxid. Med. Cell Longev..

[76] Li Y., Li X.L., Lai C.J., Wang R.S., Kang L.P., Ma T., Zhao Z.H., Gao W., Huang L.Q. (2019). Functional characterization of three flavonoid glycosyltransferases from *Andrographis paniculata*. R. Soc. Open Sci..

[77] Matsuda T., Kuroyanagi M., Sugiyama S., Umehara K., Ueno A., Nishi K. (1994). Cell differentiation-inducing diterpenes from *Andrographis paniculata* Nees. Chem. Pharm. Bull..

[78] Sani D., Khatab N.I.O., Kirby B.P., Yong A., Hasan S., Basri H., Stanslas J. (2019). A standardised. J. Adv. Res..

[79] Bera R., Ahmed S.K., Sarkar L., Sen T., Karmakar S. (2014). Pharmacokinetic analysis and tissue distribution of andrographolide in rat by a validated LC-MS/MS method. Pharm. Biol..

[80] Kumar G., Singh D., Tali J.A., Dheer D., Shankar R. (2020). Andrographolide: Chemical modification and its effect on biological activities. Bioorganic Chem..

[81] Zhang H., Li S., Si Y., Xu H. (2021). Andrographolide and its derivatives: Current achievements and future perspectives. Eur. J. Med. Chem..

[82] Wong S.Y., Tan M.G., Banks W.A., Wong W.S., Wong P.T., Lai M.K. (2016). Andrographolide attenuates LPS-stimulated up-regulation of C-C and C-X-C motif chemokines in rodent cortex and primary astrocytes. J. Neuroinflammation.

[83] Wong S.Y., Chan S.J., Wong W.S., Wong P.T., Lai M.K. (2014). Andrographolide attenuates interleukin-1β-stimulated upregulation of chemokine CCL5 and glial fibrillary acidic protein in astrocytes. Neuroreport.

[84] Das S., Mishra K.P., Ganju L., Singh S.B. (2017). Andrographolide–A promising therapeutic agent, negatively regulates glial cell derived neurodegeneration of prefrontal cortex, hippocampus and working memory impairment. J. Neuroimmunol..

[85] Adedayo B.C., Jesubowale O.S., Adebayo A.A., Oboh G. (2021). Effect of *Andrographis paniculata* leaves extract on neurobehavioral and biochemical indices in scopolamine-induced amnesic rats. J. Food Biochem..

[86] Vallée A. (2022). Neuroinflammation in Schizophrenia: The Key Role of the WNT/β-Catenin Pathway. Int. J. Mol. Sci..

[87] Hurley L.L., Tizabi Y. (2013). Neuroinflammation, neurodegeneration, and depression. Neurotox. Res..

[88] Wang X., Liu J., Dai Z., Sui Y. (2021). Andrographolide improves PCP-induced schizophrenia-like behaviors through blocking interaction between NRF2 and KEAP1. J. Pharmacol. Sci..

[89] Geng J., Liu J., Yuan X., Liu W., Guo W. (2019). Andrographolide triggers autophagy-mediated inflammation inhibition and attenuates chronic unpredictable mild stress (CUMS)-induced depressive-like behavior in mice. Toxicol. Appl. Pharmacol..

[90] Adedayo B.C., Ogunsuyi O.B., Akinniyi S.T., Oboh G. (2022). Effect of. Drug Chem. Toxicol..

[91] Iruretagoyena M.I., Tobar J.A., González P.A., Sepúlveda S.E., Figueroa C.A., Burgos R.A., Hancke J.L., Kalergis A.M. (2005). Andrographolide interferes with T cell activation and reduces experimental autoimmune encephalomyelitis in the mouse. J. Pharmacol. Exp. Ther..

[92] Zhang J., Zheng Y., Zhao Y., Zhang Y., Liu Y., Ma F., Wang X., Fu J. (2021). Andrographolide ameliorates neuroinflammation in APP/PS1 transgenic mice. Int. Immunopharmacol..

[93] Lu J., Ma Y., Wu J., Huang H., Wang X., Chen Z., Chen J., He H., Huang C. (2019). A review for the neuroprotective effects of andrographolide in the central nervous system. Biomed. Pharmacother..

[94] Dey A., Chen R., Li F., Maitra S., Hernandez J.F., Zhou G.C., Vincent B. (2021). Synthesis and Characterization of Andrographolide Derivatives as Regulators of βAPP Processing in Human Cells. Molecules.

[95] Seo J.Y., Pyo E., An J.P., Kim J., Sung S.H., Oh W.K. (2017). Andrographolide Activates Keap1/Nrf2/ARE/HO-1 Pathway in HT22 Cells and Suppresses Microglial Activation by A. Mediat. Inflamm..

[96] Yang S.L., Kuo F.H., Chen P.N., Hsieh Y.H., Yu N.Y., Yang W.E., Hsieh M.J., Yang S.F. (2017). Andrographolide suppresses the migratory ability of human glioblastoma multiforme cells by targeting ERK1/2-mediated matrix metalloproteinase-2 expression. Oncotarget.

[97] Xu Y., Tang D., Wang J., Wei H., Gao J. (2019). Neuroprotection of Andrographolide Against Microglia-Mediated Inflammatory Injury and Oxidative Damage in PC12 Neurons. Neurochem. Res..

[98] Hooper C., Killick R., Lovestone S. (2008). The GSK3 hypothesis of Alzheimer’s disease. J. Neurochem..

[99] Tapia-Rojas C., Schüller A., Lindsay C.B., Ureta R.C., Mejías-Reyes C., Hancke J., Melo F., Inestrosa N.C. (2015). Andrographolide activates the canonical Wnt signalling pathway by a mechanism that implicates the non-ATP competitive inhibition of GSK-3β: Autoregulation of GSK-3β in vivo. Biochem. J..

[100] Arredondo S.B., Valenzuela-Bezanilla D., Mardones M.D., Varela-Nallar L. (2020). Role of Wnt Signaling in Adult Hippocampal Neurogenesis in Health and Disease. Front. Cell Dev. Biol..

[101] Esquerda-Canals G., Montoliu-Gaya L., Güell-Bosch J., Villegas S. (2017). Mouse Models of Alzheimer’s Disease. J. Alzheimers Dis..

[102] Castro-Fuentes R., Socas-Pérez R. (2013). Octodon degus: A strong attractor for Alzheimer research. Basic. Clin. Neurosci..

[103] Varela-Nallar L., Arredondo S.B., Tapia-Rojas C., Hancke J., Inestrosa N.C. (2015). Andrographolide Stimulates Neurogenesis in the Adult Hippocampus. Neural Plast..

[104] Serrano F.G., Tapia-Rojas C., Carvajal F.J., Hancke J., Cerpa W., Inestrosa N.C. (2014). Andrographolide reduces cognitive impairment in young and mature AβPPswe/PS-1 mice. Mol. Neurodegener..

[105] Rivera D.S., Lindsay C., Codocedo J.F., Morel I., Pinto C., Cisternas P., Bozinovic F., Inestrosa N.C. (2016). Andrographolide recovers cognitive impairment in a natural model of Alzheimer’s disease (*Octodon degus*). Neurobiol. Aging.

[106] Lindsay C.B., Zolezzi J.M., Rivera D.S., Cisternas P., Bozinovic F., Inestrosa N.C. (2020). Andrographolide Reduces Neuroinflammation and Oxidative Stress in Aged Octodon degus. Mol. Neurobiol..

[107] Fava A., Colica C., Plastino M., Messina D., Cristiano D., Opipari C., Vaccaro A., Gorgone G., Bosco F., Fratto A. (2017). Cognitive impairment is correlated with insulin resistance degree: The “PA-NICO-study”. Metab. Brain Dis..

[108] Han R., Liang J., Zhou B. (2021). Glucose Metabolic Dysfunction in Neurodegenerative Diseases-New Mechanistic Insights and the Potential of Hypoxia as a Prospective Therapy Targeting Metabolic Reprogramming. Int. J. Mol. Sci..

[109] Yan X., Hu Y., Wang B., Wang S., Zhang X. (2020). Metabolic Dysregulation Contributes to the Progression of Alzheimer’s Disease. Front. Neurosci..

[110] Gonzalez P., Sabater L., Mathieu E., Faller P., Hureau C. (2022). Why the Ala-His-His Peptide Is an Appropriate Scaffold to Remove and Redox Silence Copper Ions from the Alzheimer’s-Related Aβ Peptide. Biomolecules.

[111] Cunnane S.C., Sieber C.C., Swerdlow R.H., Cruz-Jentoft A.J. (2021). Mild cognitive impairment: When nutrition helps brain energy rescue-a report from the EuGMS 2020 Congress. Eur. Geriatr. Med..

[112] Bosco D., Plastino M., Bosco F., Consoli A., Labate A., Pirritano D., Consoli D., Fava A. (2011). Bell’s palsy: A manifestation of prediabetes?. Acta Neurol. Scand..

[113] Patel R., Kaur K., Singh S. (2021). Protective effect of andrographolide against STZ induced Alzheimer’s disease in experimental rats: Possible neuromodulation and Aβ. Inflammopharmacology.

[114] Thakur A.K., Rai G., Chatterjee S.S., Kumar V. (2016). Beneficial effects of an *Andrographis paniculata* extract and andrographolide on cognitive functions in streptozotocin-induced diabetic rats. Pharm. Biol..

[115] Gherardelli C., Cisternas P., Gutiérrez J., Martinez M., Inestrosa N.C. (2021). Andrographolide restores glucose uptake in rat hippocampal neurons. J. Neurochem..

[116] Stoker T.B., Greenland J.C. (2018). Parkinson’s Disease: Pathogenesis and Clinical Aspects.

[117] Pajares M., Rojo A.I., Manda G., Boscá L., Cuadrado A. (2020). Inflammation in Parkinson’s Disease: Mechanisms and Therapeutic Implications. Cells.

[118] Wang T., Liu B., Zhang W., Wilson B., Hong J.S. (2004). Andrographolide reduces inflammation-mediated dopaminergic neurodegeneration in mesencephalic neuron-glia cultures by inhibiting microglial activation. J. Pharmacol. Exp. Ther..

[119] Ketterman A.J., Wongtrakul J., Saisawang C. (2020). Phytochemical andrographolide modulates NF-κB and JNK in human neuroblastoma SH-SY5Y cells, a cell model for Parkinson’s disease. Heliyon.

[120] Ahmed S., Kwatra M., Ranjan Panda S., Murty U.S.N., Naidu V.G.M. (2021). Andrographolide suppresses NLRP3 inflammasome activation in microglia through induction of parkin-mediated mitophagy in in-vitro and in-vivo models of Parkinson disease. Brain Behav. Immun..

[121] Zhang Z., Lai D., Wang L., Yu P., Zhu L., Guo B., Xu L., Zhou L., Sun Y., Lee S.M. (2014). Neuroprotective effects of the andrographolide analogue AL-1 in the MPP⁺/MPTP-induced Parkinson’s disease model in vitro and in mice. Pharmacol. Biochem. Behav..

[122] Han J., Hyun J., Park J., Jung S., Oh Y., Kim Y., Ryu S.H., Kim S.H., Jeong E.I., Jo D.G. (2021). Aberrant role of pyruvate kinase M2 in the regulation of gamma-secretase and memory deficits in Alzheimer’s disease. Cell Rep..

[123] An Y., Varma V.R., Varma S., Casanova R., Dammer E., Pletnikova O., Chia C.W., Egan J.M., Ferrucci L., Troncoso J. (2018). Evidence for brain glucose dysregulation in Alzheimer’s disease. Alzheimers Dement..

[124] Kumar V., Kim S.H., Bishayee K. (2022). Dysfunctional Glucose Metabolism in Alzheimer’s Disease Onset and Potential Pharmacological Interventions. Int. J. Mol. Sci..

[125] Pluta R. (2021). Cerebral Ischemia.

[126] Yen T.L., Hsu W.H., Huang S.K., Lu W.J., Chang C.C., Lien L.M., Hsiao G., Sheu J.R., Lin K.H. (2013). A novel bioactivity of andrographolide from *Andrographis paniculata* on cerebral ischemia/reperfusion-induced brain injury through induction of cerebral endothelial cell apoptosis. Pharm. Biol..

[127] Chan S.J., Wong W.S., Wong P.T., Bian J.S. (2010). Neuroprotective effects of andrographolide in a rat model of permanent cerebral ischaemia. Br. J. Pharmacol..

[128] Chern C.M., Liou K.T., Wang Y.H., Liao J.F., Yen J.C., Shen Y.C. (2011). Andrographolide inhibits PI3K/AKT-dependent NOX2 and iNOS expression protecting mice against hypoxia/ischemia-induced oxidative brain injury. Planta Med..

[129] Yen T.L., Chen R.J., Jayakumar T., Lu W.J., Hsieh C.Y., Hsu M.J., Yang C.H., Chang C.C., Lin Y.K., Lin K.H. (2016). Andrographolide stimulates p38 mitogen-activated protein kinase-nuclear factor erythroid-2-related factor 2-heme oxygenase 1 signaling in primary cerebral endothelial cells for definite protection against ischemic stroke in rats. Transl. Res..

[130] Shang Y.X., Shen C., Stub T., Zhu S.J., Qiao S.Y., Li Y.Q., Wang R.T., Li J., Liu J.P. (2022). Adverse Effects of Andrographolide Derivative Medications Compared to the Safe use of Herbal Preparations of. Front. Pharmacol..

